# Supplementary material for: A brainstem circuit for gravity-guided vertical navigation
Source: bioRxiv. 2024 Mar 13:2024.03.12.584680. Preprint. [Version 1] doi: 10.1101/2024.03.12.584680 (PMC10980031; doi:10.1101/2024.03.12.584680)
Supplement: Supplement 1 [file NIHPP2024.03.12.584680v1-supplement-1.pdf]

bioRxiv preprint doi: https://doi.org/10.1101/2024.03.12.584680; this version posted March 13, 2024. The copyright holder for this preprint (which was not certified by peer review) is the author/funder, who has granted bioRxiv a license to display the preprint in perpetuity. It is made available under aCC-BY 4.0 International license.

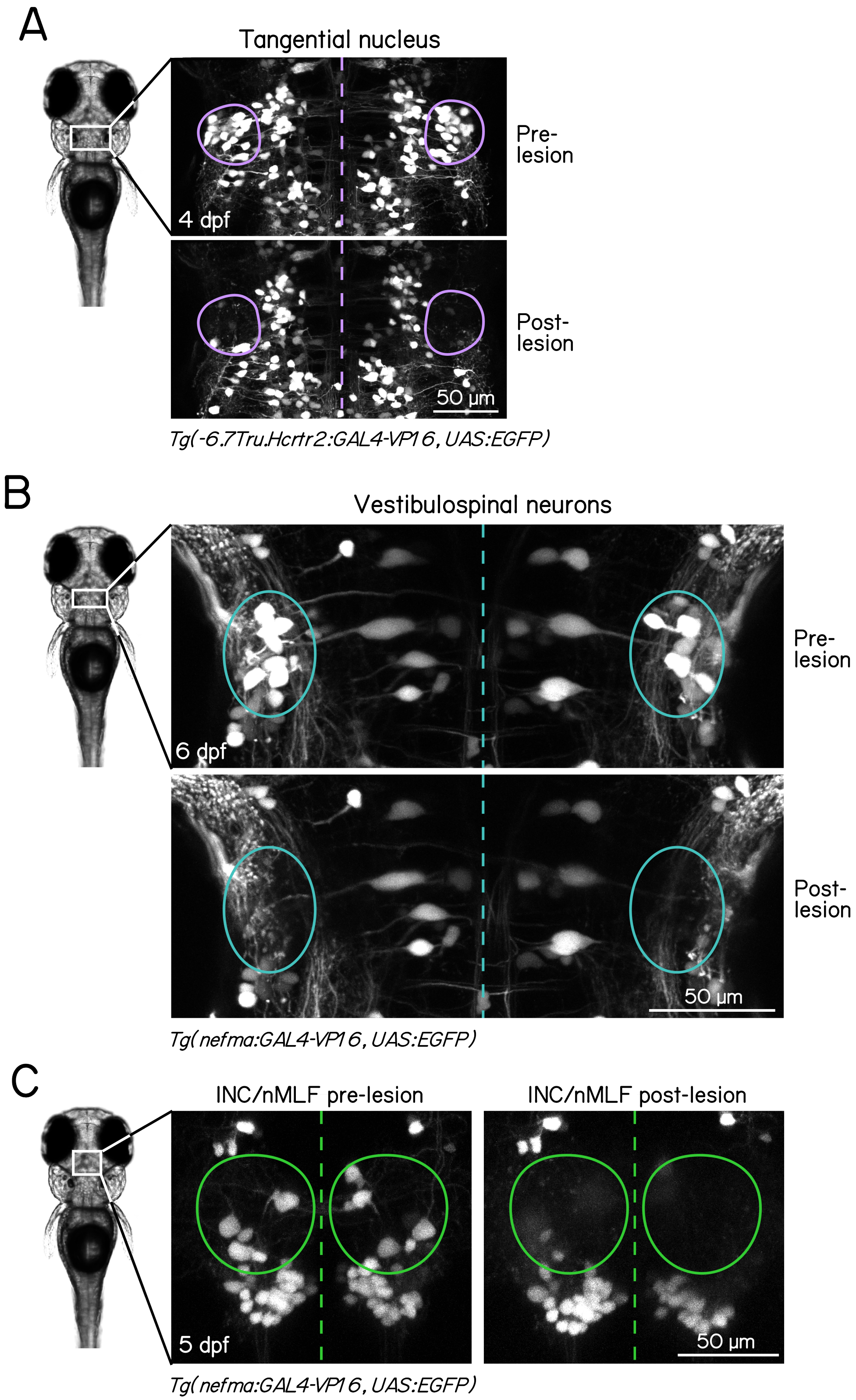

Figure S1: Example larvae before and after photoablation. **(A)** Before and after lesions of the tangential vestibular nucleus (circled) in a 4 dpf larvae. Scale bar: 50 µm. **(B)** Before and after lesions of the vestibulospinal nucleus (circled) in a 6 dpf larvae. Scale bar: 50 µm. **(C)** Before and after lesions of large neurons in the interstitial nucleus of Cajal/the nucleus of the medial longitudinal fasciculus (INC/nMLF, circled) in a 5 dpf larvae. Scale bar: 50 µm.

bioRxiv preprint doi: https://doi.org/10.1101/2024.03.12.584680; this version posted March 13, 2024. The copyright holder for this preprint (which was not certified by peer review) is the author/funder, who has granted bioRxiv a license to display the preprint in perpetuity. It is made available under aCC-BY 4.0 International license.

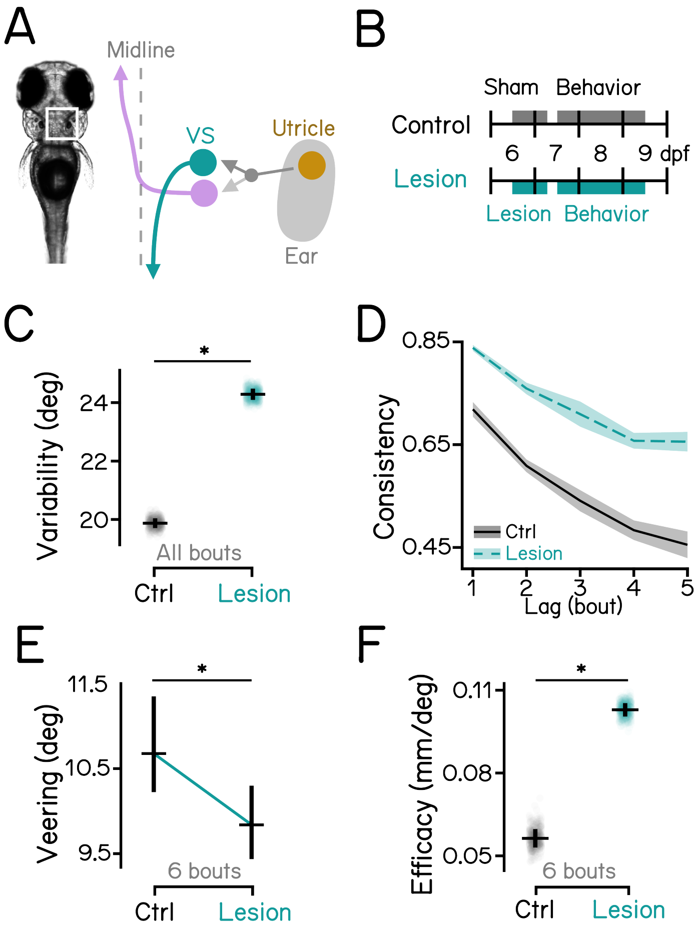

Figure S2: Lesions of vestibulospinal neurons increase postural variability but stabilize veering, improving vertical navigation. **(A)** Schematic view of the inner-ear utricular otolith and the vestibular pathways in the hindbrain of zebrafish. Utricle: utricular otoliths (yellow); VS: vestibulospinal neurons (cyan). **(B)** Diagrams of experimental procedures for lesions of the vestibulospinal nucleus and behavioral assays. See Figure S1B for examples of lesions. **(C)** Swim direction variability compared between vestibulospinal-lesioned larvae and controls. Bootstrapped MAD are plotted as data points with error bars showing standard deviations. n = 18106/17758 bouts from 79/97 fish for controls/lesions. $P_{bootstrap}$ = 3.87e-70. **(D)** Swim direction consistency plotted as a function of the number of bouts in the sequence. Shaded bands indicate standard deviations of the slope estimated using bootstrapping. **(E)** Veering through 6 consecutive bouts plotted in median with 95% confidence intervals. n = 1471/1076 6-bout series for controls/lesions. $P_{median\text{-}test}$ = 1.88e-2. **(F)** Depth change efficacy plotted with bootstrapped slopes plotted as data points and error bars showing standard deviations. $P_{bootstrap}$ = 1.17e-27. See also Table 2 for statistics.

bioRxiv preprint doi: https://doi.org/10.1101/2024.03.12.584680; this version posted March 13, 2024. The copyright holder for this preprint (which was not certified by peer review) is the author/funder, who has granted bioRxiv a license to display the preprint in perpetuity. It is made available under aCC-BY 4.0 International license.

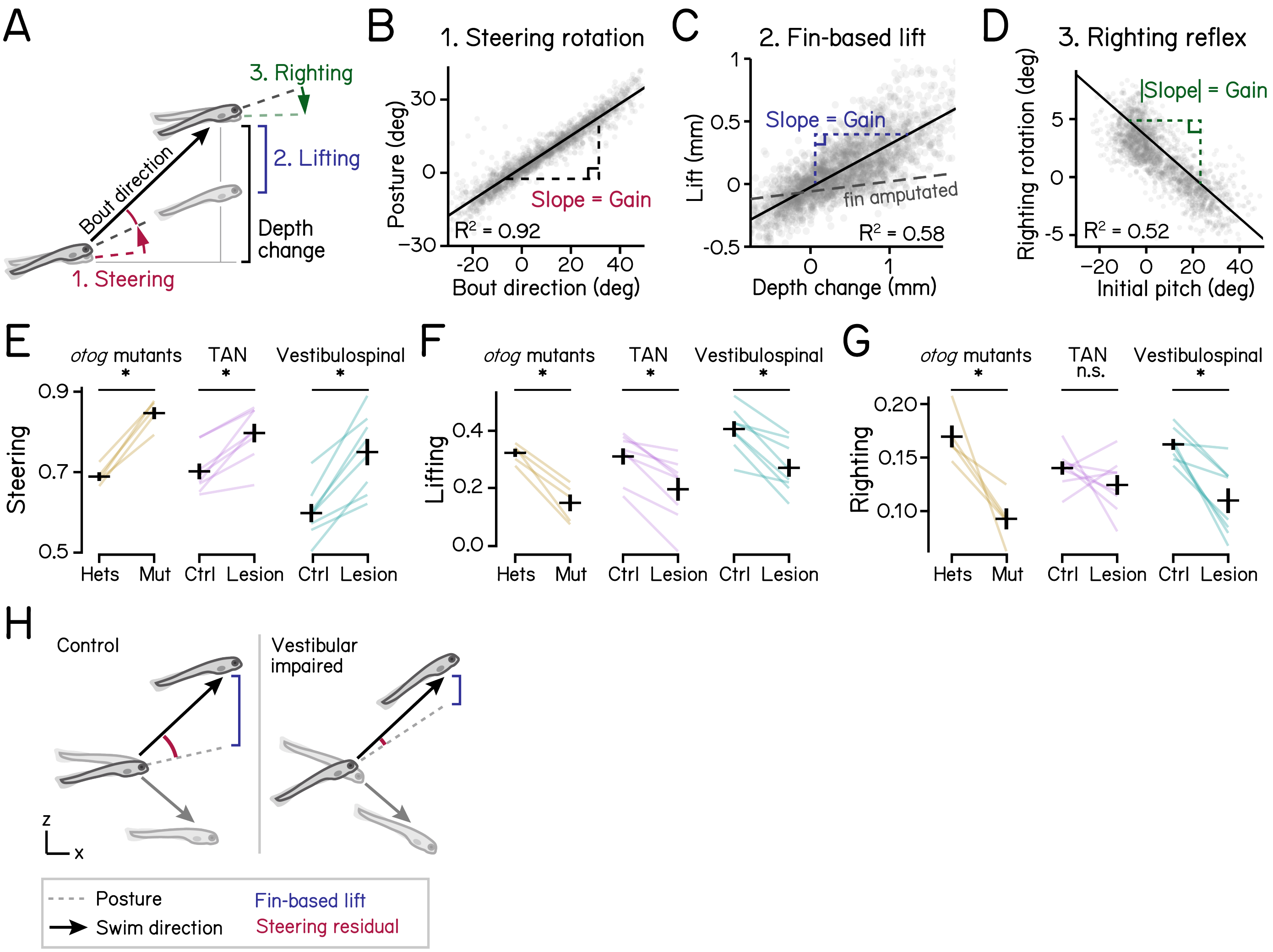

Figure S3: Vestibular contribution to swim kinematics. **(A)** Schematic diagram showing steering, lifting, and righting during a swim bout. Larvae steer toward targeted direction during acceleration (red arrow), use pectoral fins to assist in depth changes (blue), and restore posture to horizontal during deceleration (green arrow). Z displacement generated by lifting (blue) is estimated by subtracting theoretical displacement in depth, calculated from the head direction and x distance, from the total depth change. **(B)** Steering gain is defined as the slope of the best fit line of posture at the time of the peak speed vs. swim direction. n = 121,979 bouts from 537 fish. **(C)** Lift gain is defined as the slope of the best fit line of estimated lift vs. depth change of the swim bout. Pectoral-fin amputation reduces lift (dashed line). n = 33,491/28,604 bouts from 74/78 fish for control/fin-amputated. **(D)** Righting gain is defined as the numeric inversion of the slope of the best fit line of rotation during deceleration vs. initial posture. n = 121,979 bouts from 537 fish. **(E)** Steering gain of vestibular-impaired larvae vs. controls. *otog* mutation: $P_{t\text{-}test}$ =2.33e-5; tangential lesions: $P_{t\text{-}test}$ = 7.41e-3; vestibulospinal lesions: $P_{t\text{-}test}$ = 2.32e-3. N = 5/8/8 experimental repeats for *otog*/tangential lesions/ vestibulospinal lesions. Same as follows. **(F)** Lifting gain of vestibular-impaired larvae vs. controls. *otog* mutation: $P_{t\text{-}test}$ = 7.09e-4; tangential lesions: $P_{t\text{-}test}$ = 3.57e-2; vestibulospinal lesions: $P_{t\text{-}test}$ = 5.90e-3. **(G)** Righting gain of vestibular-impaired larvae vs. controls. *otog* mutation: $P_{t\text{-}test}$ = 6.76e-4; tangential lesions: $P_{t\text{-}test}$ = .180; vestibulospinal lesions: $P_{t\text{-}test}$ = 1.00e-3.
(H) Summary of effects of vestibular perturbations on bout kinematics. Vestibular-impaired fish swim with more eccentric posture and less fin-based lift. Abbreviation: TAN, tangential. See also Table 1 for parameter definitions and Table 2 for statistics.
